# Cardiac MIBG Scintigraphy in Neurodegenerative Parkinsonism: Limitations in Clinical Practice

**DOI:** 10.1002/mdc3.70656

**Published:** 2026-04-26

**Authors:** Frank Jagusch, Elena Redl, Christoph Theyer, Atbin Djamshidian, Alessandra Fanciulli, Werner Poewe, Philipp Mahlknecht, Beatrice Heim, Klaus Seppi, Florian Krismer

**Affiliations:** ^1^ Department of Neurology Innsbruck Medical University Innsbruck Austria

**Keywords:** ^123^I‐meta‐iodobenzylguanidine (^123^I‐MIBG) myocardial scintigraphy, clinical applications, multiple system atrophy, Parkinson's disease, progressive supranuclear palsy

## Abstract

**Background:**

Reduced cardiac uptake on ^123^Iodine‐metaiodobenzylguanidine (MIBG) scintigraphy is a valuable tool for differentiating neurodegenerative parkinsonism but interpretation can be difficult due to comorbidities and drug‐tracer interactions.

**Objectives:**

This study investigated factors limiting the clinical applicability of MIBG scintigraphy in everyday clinical practice.

**Methods:**

Patient records (2021–2022) were retrospectively reviewed, independent of whether MIBG scintigraphy was performed, for comorbidities and concomitant medication confounding its interpretation.

**Results:**

Six hundred ninety five patients (640 Parkinson's disease, 22 multiple system atrophy, 33 progressive supranuclear palsy) were included. Factors potentially influencing cardiac MIBG binding were present in 55.3% with 18.1% exhibiting multiple factors. Medications with known drug‐tracer interactions were found in 46.9% and comorbidities associated with reduced cardiac MIBG uptake in 20.9%.

**Conclusions:**

The clinical utility of MIBG scintigraphy is frequently restricted by medications and comorbidities highlighting the need of careful planning in clinical practice.

Cardiac scintigraphy with the norepinephrine analogue ^123^Iodine‐metaiodobenzylguanidine (MIBG) is a nuclear imaging technique capable of assessing noradrenergic cardiac innervation in vivo. The cardiac uptake of MIBG is semi‐quantitatively analyzed using the Heart‐to‐Mediastinum (H/M) ratio in early and delayed images.^1^, ^2^ While in Parkinson's disease (PD) and dementia with Lewy bodies, sympathetic denervation of the heart is a common finding, the H/M ratio is usually normal or only mildly reduced in patients with multiple system atrophy (MSA) and progressive supranuclear palsy (PSP).^1^, ^3^, ^4^, ^5^ Consequently, MIBG scintigraphy is recognized as an accurate diagnostic tool for differentiating neurodegenerative parkinsonian diseases, with reported sensitivity of up to 95.5% and specificity of up to 91.0%.^6^, ^7^, ^8^, ^9^ Consequently, abnormal findings are included as a supportive feature in the diagnostic criteria for PD^10^ and as an exclusion criterion for MSA.^11^ Cardiac ^18^F‐dopamine positron emission tomography is a reliable alternative to MIBG scintigraphy, its clinical use is limited by its even more restricted availability.^12^, ^13^ However, the application and interpretation of MIBG scintigraphy have inherent limitations, such as limited availability and high costs with lacking cost‐effectiveness, and its susceptibility to false‐positive (ie, reduced cardiac tracer uptake) results caused by structural heart abnormalities (eg, heart failure [HF], ischemic cardiomyopathy [ICM]), concurrent peripheral nervous system disease (eg, polyneuropathies [PN]) as well as drug‐tracer interactions (eg, antidepressants; a comprehensive list of medication that may affect MIBG scintigraphy interpretation is available in the ASNC imaging guidelines^14^).^1^, ^4^, ^14^, ^15^, ^16^, ^17^, ^18^, ^19^, ^20^ In the context of using cardiac MIBG imaging as supportive criterion for MSA, medications affecting noradrenaline transporter and vesicular storage, structural heart disease, and common causes of small fiber neuropathies, such as diabetes mellitus, must be excluded.^10^, ^11^ The primary objective of this analysis was to assess the frequency of influencing factors that can affect the interpretation of MIBG scintigraphy results in an unselected cohort of patients with Parkinsonism in a tertiary referral center.

## Methods

This retrospective study was approved by the ethics committee of the Medical University of Innsbruck (1368/2022). Patients with parkinsonism attending the movement disorder outpatient clinic at the Medical University of Innsbruck between July 1, 2021, and June 30, 2022, were included and patients with a diagnosis of a neurodegenerative parkinsonism (ie, PD, MSA or PSP) at the last visit were considered for analyses. Demographics and clinical information were tabulated from patient records. In addition, paper as well as digital case records were screened for a history of diseases impacting the reliability of MIBG scintigraphy. Moreover, any additional investigations, ie, echocardiography or nerve conduction studies (NCS), were reviewed to ascertain the absence of conditions associated with reduced cardiac noradrenergic innervation. The medication history of the subjects was also reviewed, paying particular attention to any medications known to interact with MIBG tracer uptake. These influencing factors were categorized into modifiable factors (ie, intake of reserpine, tricyclic antidepressants (TCA), selective serotonin and norepinephrine reuptake inhibitors (SSRI/SNRI), calcium channel blockers (CCB), sympathomimetics and labetolol), and non‐modifiable factors including comorbidities such as PN, HF, ICM or heart transplantation (HTx).

Final clinical diagnosis not meeting any established diagnostic criteria or insufficient case documentation (ie, incomplete or lack of documented medication and comorbidities) were exclusion criteria.

Statistical analysis was conducted using IBM SPSS Statistics Version 29.0.2.0. Frequency distributions were used to describe nominal and ordinal variables. Differences between the diagnostic groups were analyzed based on data distribution, with non‐parametric tests applied where appropriate. A significance level of *p* ≤ 0.05 was considered statistically significant; because of the exploratory nature of this study, *p* values were not corrected for multiple testing.

## Results

Patients’ characteristics and clinical information are summarized in Table 1. In total, 695 patients (38.7% female) met the inclusion criteria and had sufficient case documentation. Of these, 640 (92.1%) were diagnosed with PD, 22 (3.2%) with MSA, 33 (4.7%) with PSP. Although PSP patients were in general older, age differences were not significant between groups at first presentation (*p* = 0.060). The disease duration was significant different between groups with PD patients having longer disease durations compared to MSA and PSP patients (*p* < 0.001).

**TABLE 1:** Demographics of the cohort

	Total			
	PD	MSA	PSP	*p* value
n (%)	695			‐
	640 (92.1)	22 (3.2)	33 (4.7)	
Sex (m:f; %)	426:269 (61.3:38.7)			0.022^a^
	394:246	8:14	24:9	
Age at onset (years; median + IQR)	62.0 (16.0)			0.006^b^
	62.0 (17.0)	62.0 (14.5)	69.0 (11.5)	
Age at first visit (year; median + IQR)	65.0 (15.0)			0.060^b^
	65.0 (15.3)	66.0 (12.0)	71.0 (11.5)	
Age at last visit (years; median + IQR)	72.0 (15.0)			0.015^b^
	72.0 (16.0)	67.0 (13.5)	75.0 (10.0)	
Disease duration (years; median + IQR)	7.5 (9.0)			< 0.001^b^
	8.0 (9.0)	5.0 (4.0)	5.0 (4.5)	
Follow‐up period (years; median + IQR)	4.0 (7.0)			< 0.001^b^
	4.0 (7.0)	1.0 (3.0)	3.0 (3.0)	
Hoehn and Yahr stage (median + IQR)	2.0 (2.0)			< 0.001^b^
Stage 1 (%)	120 (18.8)	1 (4.5)	0 (0.0)	‐
Stage 2 (%)	250 (39.1)	0 (0.0)	1 (3.0)	‐
Stage 2.5 (%)	2 (0.3)	0 (0.0)	0 (0.0)	‐
Stage 3 (%)	93 (14.5)	1 (4.5)	3 (9.1)	‐
Stage 4 (%)	67 (10.5)	5 (22.7)	4 (27.3)	‐
Stage 5 (%)	104 (16.3)	15 (68.2)	20 (60.6)	‐
MIBG performed (%)	10 (1.4)			0.038^c^
	7 (1.1)	1 (4.5)	2 (6.1)	

Abbreviations: f, female; IQR, interquartile range; m, male.

^a^ Disease duration defined as from symptom onset to last visit, follow‐up period defined as first to last visit; group comparison using *χ* ^2^ test.

^b^ Kruskal‐Wallis test.

^c^ Fisher–Freeman–Halton exact test.

Cardiac MIBG scintigraphy was performed in 10 patients (7 PD, 1 MSA, 2 PSP; *p* = 0.038) with reduced MIBG uptake observed in 70% (5 PD, 2 PSP; *p =* 0.358; sensitivity 71.4%, specificity 33.3%). Among these, 85.7% showed at least one factor potentially impacting the reliability of a MIBG scintigraphy reading, while non‐modifiable factors were present in both patients with a final diagnosis of PSP. Overall, 55.3% of patients (345 PD, 14 MSA, 25 PSP; *p* = 0.035) had at least one influencing factor, with 18.1% presenting multiple contributing factors. A total of 86 patients (12.4%) showed both modifiable and non‐modifiable factors (*p* = 0.423). Isolated non‐modifiable factors were present in 8.5% (*p* = 0.322), whereas isolated modifiable factors were found in 34.5% (*p* = 0.326). Among the medications that interact with the MIBG tracer, SSRI/SSNI were most observed with 34.8% (*p* = 0.226), followed by sympathomimetics in 10.8% (*p* = 0.045), CCB in 8.3% (*p* = 0.127) and TCA in 1.0% (*p* = 0.440) of the cases. While 34.5% (*p* = 0.326) showed only one of the modifiable factors, multiple medications were reported in 7.6% (*p* = 0.078). Comorbidities (ie, non‐modifiable factors) confounding MIBG scintigraphy were found in 20.9% (*p* = 0.215) of the patients. 12.7% (*p* = 0.114) of all case records showed PN, which had been confirmed through NCS in 86.3% (*p* = 0.195) of the cases. While 12.7% (*p* = 0.520) showed risk factors for ICM such as coronary artery disease and echocardiographic wall motion abnormalities, only 14 patients (*p* = 1.000) had a confirmed diagnosis of ICM. HF was reported in 8.5% (*p* = 0.671), with echocardiographic confirmation in 78.0% (*p* = 0.688). Multiple non‐modifiable factors were present in 22 cases (*p* = 0.075). There was no significant difference in the distribution of influencing factors between sexes (*p* = 0.318). Influencing factors impacting MIBG readouts are summarized in Table 2.

**TABLE 2:** Modifiable and non‐modifiable factors impacting MIBG readouts

		Total			
		PD	MSA	PSP	*p* value
Influencing factors overall	IF overall	384 (55.3)			0.035^a^
		345 (53.9)	14 (63.6)	25 (75.8)	
	IF overall, multiple	126 (18.1)			0.310^a^
		112 (17.5)	5 (22.7)	9 (27.3)	
	IF modifiable and non‐modifiable	86 (12.4)			0.423^b^
		77 (12.0)	3 (13.6)	6 (18.2)	
Influencing factors non‐modifiable	IF non‐modifiable	145 (20.9)			0.215^b^
		130 (20.3)	4 (18.2)	11 (33.3)	
	IF non‐modifiable, isolated	59 (8.5)			0.322^b^
		53 (8.3)	1 (0.1)	5 (15.2)	
	IF non‐modifiable, multiple	22 (3.2)			0.075^b^
		18 (2.8)	1 (4.5)	3 (9.1)	
	Polyneuropathy (%)	88 (12.7)			0.114^b^
		77 (12.0)	3 (13.6)	8 (24.2)	
	NCS confirmed PN (%)	76 (86.3)			0.195^b^
		65 (84.4)	3 (100.0)	8 (100.0)	
	Ischemic cardiomyopathy (%)	14 (2.0)			1.000^b^
		14 (2.2)	0 (0.0)	0 (0.0)	
	Risk factors for ICM (%)	88 (12.7)			0.520^b^
		84 (13.1)	1 (4.5)	3 (9.1)	
	Heart failure (%)	59 (8.5)			0.671^b^
		53 (8.3)	2 (9.1)	4 (12.1)	
	Echocardiography confirmed heart failure (%)	46 (78.0)			0.688^b^
		45 (84.9)	1 (50.0)	0 (0.0)	
	Heart transplantation (%)	0 (0.0)			‐
		0 (0.0)	0 (0.0)	0 (0.0)	
Influencing factors modifiable	IF modifiable	326 (46.9)			0.127^a^
		293 (45.8)	13 (59.1)	20 (60.6)	
	IF modifiable, isolated	240 (34.5)			0.326^a^
		216 (33.8)	10 (45.5)	14 (42.4)	
	IF modifiable, multiple	53 (7.6)			0.078^b^
		45 (7.0)	3 (13.6)	5 (15.6)	
	Reserpine (%)	0 (0.0)			‐
		0 (0.0)	0 (0.0)	0 (0.0)	
	Tricyclic antidepressants (%)	7 (1.0)			0.440^b^
		6 (0.9)	0 (0.0)	1 (3.0)	
	SSRI/SNRI (%)	242 (34.8)			0.226^a^
		217 (33.9)	10 (45.5)	15 (45.5)	
	Sympathomimetics (%)	75 (10.8)			0.045^b^
		65 (10.2)	6 (27.3)	4 (12.1)	
	Calcium channel blockers (%)	58 (8.3)			0.127^b^
		53 (8.3)	0 (0.0)	5 (15.2)	
	Labetolol (%)	0 (0.0)			‐
		0 (0.0)	0 (0.0)	0 (0.0)	

Abbreviations: ICM, ischemic cardiomyopathy; IF, influencing factor; NCS, nerve conducting study; PN, polyneuropathy; SSRI/SSNI, selective serotonin/norepinephrine reuptake inhibitors.

^a^ Group comparison using *χ* ^2^ test.

^b^ Fisher–Freeman–Halton exact test.

## Discussion

Although cardiac MIBG scintigraphy reliably discriminates between different neurodegenerative parkinsonian diseases, its clinical applicability is frequently constrained by availability, concomitant medication use and comorbidities affecting the reliability of image interpretation.

In the present study, we retrospectively analyzed the prevalence of factors negatively impacting the diagnostic yield in a cohort of 695 patients (640 PD, 22 MSA, 33 PSP). Overall, 55.3% (53.9% PD, 63.6% MSA, 75.8% PSP) were identified as having at least one factor potentially affecting the interpretation of cardiac MIBG scintigraphy. Antidepressant use, reported in 36% of cases, was the most common single factor. Depression is a common non‐motor symptom in parkinsonian disorders. Its prevalence is estimated to be around 30–40% in PD patients, with even higher prevalence rates in MSA (43%) and PSP (50%).^21^, ^22^, ^23^, ^24^, ^25^ SSRI/SSNI and TCA are the most common used antidepressant drugs in parkinsonian disorders, with estimated 25% of PD patients requiring treatment with antidepressants in disease course.^21^, ^22^, ^23^, ^26^ The rate of antidepressant intake in our cohort is consistent with these numbers.

Although concomitant medication is recommended to be discontinued prior to MIBG scintigraphy and is therefore classified as modifiable, discontinuation requires usually one to several weeks and may pose potential risks and harmful effects for patients.^14^, ^18^, ^27^

The most common non‐modifiable factor was the presence of PN (12.0% PD, 13.6% MSA, 24.2% PSP). PN prevalence in the general population has been reported to be about 9%, whereas in PD patients, it is higher and ranges from 5% to 73%.^28^, ^29^, ^30^ Data on MSA and PSP are limited, although the burden of PN has previously been described as 87.5% in MSA and 66.7% in PSP, with electrophysiological confirmation of 50.0% each.^31^ In our cohort, PN was present in 13.6% of MSA and 24.2% of PSP patients, exceeding the 12.0% observed in our PD patients. Therefore, our findings align with the literature, showing higher prevalence than the general population but a lower prevalence than previously described in atypical parkinsonian syndromes. The frequency of PN in MSA patients has significant implications for clinical practice, since MIBG imaging is most useful for the differential diagnosis of MSA versus PD with PN‐associated MIBG reductions in MSA patients possibly reducing the differential diagnostic accuracy.

Previous studies suggested that PD patients are at increased risk of cardiovascular and structural heart disease, including HF and ICM, the latter being the most common cause of HF in developed countries.^32^, ^33^ In our cohort, only 2.0% (14 PD) had confirmed ICM, whereas 12.7% (13.1% PD, 4.5% MSA, 9.1% PSP) showed risk factors for ICM. HF was documented in 8.5% (8.3% PD, 9.1% MSA, 12.1% PSP) of patients. The approximate prevalence of HF in the general elderly population (>65 years) is described as about 10%, while the prevalence in PD patients is described up to 20%.^34^ Importantly, 86.3% of PN diagnoses and 78.0% of HF diagnoses have been confirmed through NCS or echocardiography, respectively. However, NCS and echocardiography were performed in only 23.9% and 20.7% of the study population.

Cardiac MIBG scintigraphy was performed in 10 patients, with reduced cardiac uptake in 70% (5 PD, 2 PSP). Diagnostic performance was limited (sensitivity 71.4%, specificity 33.3%), likely reflecting the small sample size and the fact that both PSP patients had non‐modifiable factors. Notably, one scan in a PSP patient showed only mildly reduced uptake. In addition, previous studies investigating the use of myocardial MIBG scintigraphy to differentiate parkinsonian disorders often excluded patients with potentially influencing concomitant medication or comorbidities, which may overemphasize its applicability in clinical practice and this is reflected with the low rate of performance (1.4%) of MIBG scintigraphy in our clinical cohort.^7^, ^35^

A key strength of this study is the unfiltered inclusion of all cases from our tertiary movement disorder center, allowing good generalizability of the results. However, this study has also potential limitations. These primarily arise from the retrospective study design as it carries the risk of reporting bias and inconsistent documentation in case records. Additionally, examinations to exclude comorbidities were not systematically performed. These limitations could contribute to a further underestimation of the frequency and the impact of comorbidities on the applicability of cardiac MIBG scintigraphy.

In summary, cardiac MIBG scintigraphy is a valuable method for differentiating neurodegenerative Parkinsonism. However, its clinical applicability is limited by multiple modifiable and non‐modifiable factors, requiring thorough examination planning and timely discontinuation of concomitant medication to avoid false‐positive PD diagnoses.

## Author Roles

(1) Research Project: A. Conception, B. Organization, C. Execution; (2) Statistical Analysis: A. Design, B. Execution, C. Review and Critique; (3) Manuscript Preparation: A. Writing of the First Draft, B. Review and Critique.

F.J.: 1A, 1B, 1C, 2A, 2B, 3A.

E.R.: 1A, 1B, 1C, 2A, 2B, 3A.

C.T.: 2C, 3A.

A.D.: 1A, 2C, 3B.

A.F.: 1A, 2C, 3B.

W.P.: 1A, 2C, 3B.

P.M.: 1A, 2C, 3B.

B.H.: 1A, 2C, 3B.

K.S.: 1A, 2C, 3B.

F.K.: 1A, 1B, 2A, 2C, 3A, 3B.

## Disclosures

**Ethical Compliance Statement:** This study was approved by the ethics committee of the Medical University of Innsbruck (1368/2022). Given the retrospective nature of this study at an academic medical center, informed patient consent was not necessary for this work. We confirm that we have read the Journal's position on issues involved in ethical publication and affirm that this work is consistent with those guidelines.

**Funding Sources and Conflicts of Interest:**. This research was funded in part by the Austrian Science Fund (FWF) [FG2700, DOI: 10.55776/FG27]. Open access funding provided by the Medical University Innsbruck. The authors declare that there are no conflicts of interest relevant to this work.

**Financial Disclosures for the Previous 12 Months:** F.J., E.R., C.H. declare no conflict of interest. A.D. reports honoraria from Eisai, Roche, Lilly, NovoNordisk and grants from the Austrian Science Fund (FWF). A.F. reports royalties from Springer Verlag, speaker fees and honoraria from Antag Therapeutics, American Academy of Neurology, Austrian Neurology Society, Austrian Autonomic Society, Bial, Broadview Ventures, CNSystem, Desitin, Donau Krems University, Elsevier, GE Healthcare, Healthware International, International Parkinson and Movement Disorders Society, KABEG, Medizin Forum, Medtronic, Prime Global, Sanofi, Theravance Biopharma. She reports research grants from the Medical University of Innsbruck, Mission MSA, Dr Johannes and Hertha Tuba Foundation and Austrian Exchange Program. She is an associate editor of Clinical Autonomic Research and Autonomic Neuroscience, the president of the European Federation of Autonomic Society and the president elect of the Austrian Autonomic Society. She coordinates the Disease Group on Atypical Parkinsonian Disorders of the European Reference Network for Rare Neurological Disorders and a guideline project on the symptomatic treatment of atypical Parkinsonian Syndromes for the European Academy of Neurology. W.P. reports personal fees from Alterity, AbbVie, AC Immune, BIAL, Boehringer, Britannia, Lilly, Lundbeck, Merck, Merz Pharma, Mitsubishi‐Tanabe, Neuroderm, Novo Nordisk, Roche, Sanofi, Sumitomo, Takeda, Teva, UCB and Zambon (consultancy and lecture fees in relation to clinical drug development programmes for PD and MSA) and royalties from Thieme, Wiley Blackwell, Oxford University Press and Cambridge University Press. He reports grants from Michael J. Fox Foundation (MJFF) and the European Union (EU FP7 & Horizon 2020). P.M. reports speaker's honoraria from AbbVie and a grant from Michael J. Fox Foundation (MJFF). B.H. reports honoraria from Novartis AG, BIAL, AbbVie, Merz, Stada, and grants from the Austrian science fund (FWF). K.S. reports grants from the Austrian Science Fund (FWF), Michael J. Fox Foundation (MJFF), and the International Parkinson and Movement Disorder Society, as well as personal fees from Teva, UCB, Lundbeck, AOP Orphan Pharmaceuticals AG, Stada, Novartis, Eisai, Bial, Roche, and Grünenthal. F.K. reports personal fees from AbbVie, Bial, Institut de Recherches Internationales Servier, Koneksa, Österreichische Apotheker‐Verlagsgesellschaft, Sanofi, Takeda Pharmaceuticals, and Teva. He reports grants from the Medical University Innsbruck, National Institutes of Health (NIH), and the Michael J. Fox Foundation (MJFF).

## Data Availability

The data that support the findings of this study are available from the corresponding author upon reasonable request.
